# Nuclear receptors from the ctenophore *Mnemiopsis leidyi *lack a zinc-finger DNA-binding domain: lineage-specific loss or ancestral condition in the emergence of the nuclear receptor superfamily?

**DOI:** 10.1186/2041-9139-2-3

**Published:** 2011-02-03

**Authors:** Adam M Reitzel, Kevin Pang, Joseph F Ryan, James C Mullikin, Mark Q Martindale, Andreas D Baxevanis, Ann M Tarrant

**Affiliations:** 1 Biology Department, Woods Hole Oceanographic Institution, Woods Hole, MA, USA; 2 Kewalo Marine Laboratory, Pacific Bioscience Research Center, University of Hawaii, Honolulu, HI, USA; 3 Genome Technology Branch, National Human Genome Research Institute, National Institutes of Health, Bethesda, MD, USA

## Abstract

**Background:**

Nuclear receptors (NRs) are an ancient superfamily of metazoan transcription factors that play critical roles in regulation of reproduction, development, and energetic homeostasis. Although the evolutionary relationships among NRs are well-described in two prominent clades of animals (deuterostomes and protostomes), comparatively little information has been reported on the diversity of NRs in early diverging metazoans. Here, we identified NRs from the phylum Ctenophora and used a phylogenomic approach to explore the emergence of the NR superfamily in the animal kingdom. In addition, to gain insight into conserved or novel functions, we examined NR expression during ctenophore development.

**Results:**

We report the first described NRs from the phylum Ctenophora: two from *Mnemiopsis leidyi *and one from *Pleurobrachia pileus*. All ctenophore NRs contained a ligand-binding domain and grouped with NRs from the subfamily NR2A (*HNF4*). Surprisingly, all the ctenophore NRs lacked the highly conserved DNA-binding domain (DBD). NRs from *Mnemiopsis *were expressed in different regions of developing ctenophores. One was broadly expressed in the endoderm during gastrulation. The second was initially expressed in the ectoderm during gastrulation, in regions corresponding to the future tentacles; subsequent expression was restricted to the apical organ. Phylogenetic analyses of NRs from ctenophores, sponges, cnidarians, and a placozoan support the hypothesis that expansion of the superfamily occurred in a step-wise fashion, with initial radiations in NR family 2, followed by representatives of NR families 3, 6, and 1/4 originating prior to the appearance of the bilaterian ancestor.

**Conclusions:**

Our study provides the first description of NRs from ctenophores, including the full complement from *Mnemiopsis*. Ctenophores have the least diverse NR complement of any animal phylum with representatives that cluster with only one subfamily (NR2A). Ctenophores and sponges have a similarly restricted NR complement supporting the hypothesis that the original NR was *HNF4*-like and that these lineages are the first two branches from the animal tree. The absence of a zinc-finger DNA-binding domain in the two ctenophore species suggests two hypotheses: this domain may have been secondarily lost within the ctenophore lineage or, if ctenophores are the first branch off the animal tree, the original NR may have lacked the canonical DBD. Phylogenomic analyses and categorization of NRs from all four early diverging animal phyla compared with the complement from bilaterians suggest the rate of NR diversification prior to the cnidarian-bilaterian split was relatively modest, with independent radiations of several NR subfamilies within the cnidarian lineage.

## Background

Nuclear receptors (NRs) are a class of transcription factors that regulate diverse developmental and physiological processes in animals. The characteristic domains of NRs are the DNA-binding domain (DBD), which includes a Cys-Cys zinc coordinating region, and the ligand-binding domain (LBD), a carboxy-terminal domain that binds ligands and coactivators for modulation of NR function, facilitates receptor dimerization, and contains an activation function. Current evidence has shown that NRs are restricted to animals because they are present in all metazoan phyla that have been surveyed but not in plants or fungi [1-6]. NR-like transcription factors have been identified in some fungi based on a region that has low conservation with the LBD of animal NRs; however, whether these are evolutionary related to NRs in animals is not known [7].

Genes in the NR superfamily are classified into six major families (NR 1 through NR 6; Nuclear Receptor Nomenclature Committee [8]). All six families (and many subfamilies) are represented in protostomes and deuterostomes, supporting the conclusion that NRs had diversified prior to the bilaterian ancestor [1]. Thus, characterizing NRs in species representative of the early diverging animal phyla would provide critical information that would allow for a better understanding of the evolutionary history and emergence of this superfamily. NRs have been reported from three of the four classes of cnidarians (Anthozoa [3,5], Cubozoa [9], and Hydrozoa [2]), including the full complement from the sea anemone *Nematostella vectensis *[5], two sponges [4,6,10], and one placozoan [11,12]. Phylogenetic analyses of these NRs have shown that most of these genes belong to NR family 2. The exceptions are: (1) one NR family 3 member from the placozoan *Trichoplax adhaerens*, (2) one ortholog to NR family 6 from the cnidarian *Nematostella*, and (3) three genes from *Nematostella *that form an outgroup to NR families 1 and 4. Taken together, these data suggest that the evolution of NRs may be complex and, more importantly, that early diverging metazoans are the appropriate groups to study in order to understand how and when the NR superfamily has evolved and diversified.

Among the early branching animal lineages, NRs have not been identified or described in any species from Phylum Ctenophora. The apparent phylogenetic position of ctenophores in relation to other animal phyla has varied among studies, with recent analyses placing them at the base of the animal tree [13,14], as a sister clade with other non-bilaterians to the Bilateria [15], in one clade with cnidarians [16], or as an outgroup to a clade of the Placozoa, Cnidaria, and Bilateria [17,18] (see [19] for a summary). Determining the correct phylogenetic position of ctenophores and other metazoan phyla is critical for accurately reconstructing the evolutionary history of individual gene families. Reciprocally, accumulated analyses comparing the diversity of multiple gene families among early diverging phyla may help investigators to select among the particular phylogenetic hypotheses. Characterizing the NR complement from a ctenophore species is a constructive step towards understanding the evolution of the superfamily and, at the same time, it provides some insight into the relationship of ctenophores to other metazoans. Similarly, comparing the expression of NRs in ctenophores with other species would provide data needed to assess potential conservation of the spatial and temporal expression of these transcription factors.

Here, we describe NRs from two ctenophores, the lobate *Mnemiopsis leidyi *and the tentaculate *Pleurobrachia pileus*. These two species are from different orders and, thus, represent distant lineages within the Ctenophora [20]. Additionally, for *Mnemiopsis*, we describe the intron-exon structure and the spatio-temporal developmental expression of these NRs using *in situ *hybridization. We then applied phylogenetic methods to characterize NR diversity from 12 early diverging metazoans (two sponges, two ctenophores, one placozoan, and seven cnidarians) and developed a hypothesis for the early diversification of the NR superfamily.

## Methods

### Identification and annotation of Mnemiopsis leidyi nuclear receptors

We identified candidate NRs through BLAST queries of the assembled genome and gene models for *Mnemiopsis leidyi *(physical coverage of genome approximately 50×, see [21]). For these searches we used a diverse set of full-length NRs from the sponge *Amphimedon queenslandica*, the anemone *Nematostella vectensis*, *Homo sapiens*, and *Drosophila melanogaster*. Through these similarity searches we identified two matches that were reciprocal best BLAST hits with other animal NRs, showing strong similarity to the LBD. No BLAST queries returned any ctenophore gene model or region of the genome with similarity to the DBD. TBLASTN searches of the *Mnemiopsis *genome and BLASTP searches of the proteome using only the DBD from sponge and anemone *HNF4 *resulted in weak matches that were on separate contigs from the two predicted genes from *Mnemiopsis *with high similarity to the LBD. The top ctenophore protein match using the DBD from sponge *HNF4 *(E-value = 0.027) was BLASTed to human refseq and exhibited greatest, although low, similarity to members of the LIM family. HMM searches of the ctenophore proteome failed to identify any additional matches.

For the two candidate NRs, we performed 5'- and 3'-RACE with gene specific primers, using cDNA prepared from RNA pooled from diverse developmental stages (see Additional file 1 for all primers). RACE products were cloned into pGEMT (Promega, Madison, WI, USA) and sequenced. Overlapping fragments were assembled *in silico *to produce the complete transcript. To confirm the two gene products, we amplified and sequenced the entire open reading frame for each NR. Each primer pair yielded a single product that matched the conceptually assembled fragment. We characterized gene structure for each *Mnemiopsis *NR by aligning the full length transcripts to the assembled genome. We also identified a NR in a second species of ctenophore, *Pleurobrachia pileus*, through BLAST queries of ESTs in GenBank. We assembled a full-length transcript for a single *Pleurobrachia *NR by assembling overlapping ESTs (GenBank accession numbers FP998505, FP993827, FP993707, and FP997412).

### Developmental expression of Mnemiopsis nuclear receptors

Whole-mount *in situ *hybridizations were performed as previously described [22]. A RACE product for *MlNR1 *(approximately 1,000 bp) and the full open reading frame for *MlNR2 *(1,137 bp) were used as templates to transcribe digoxigenin-labeled antisense RNA probes (Megascript Kit, Ambion, Austin, TX, USA). Developmental stages from gastrulation through the early cydippid were probed for spatial expression.

### Phylogenetic analyses of the nuclear receptor superfamily

Through a combination of literature searches and BLAST queries, we assembled a dataset of 54 NRs from early diverging animals in the phyla Ctenophora, Porifera, Placozoa, and Cnidaria (Accession numbers given in Additional file 2). We identified three additional NRs from the coral *Acropora millepora *[data from 23] which were combined with previously described NRs from this species [3]. NRs from two cnidarians (*Metridium senile *(n = 2), *Hydra magnipapillata *(n = 6)) were identified through BLAST queries against GenBank. Sequences from *Homo sapiens*, *Drosophila melanogaster*, and *Caenorhabditis elegans *from Bertrand *et al. *[1] were used as representative bilaterian sequences.

Full-length sequences for all taxa were aligned with Muscle 3.6 [24] and edited manually in the case of clear errors. For some taxa, only partial sequences were available, containing only the DBD in many cases. Due to the absence of a well-conserved DBD in the three ctenophore sequences, these proteins were manually corrected in an effort to optimize the alignment.

Maximum likelihood analyses were conducted with RAxML (version 7.0.4, [25]) and Bayesian analyses with MrBayes v.3.1.2 [26] using a JTT+G+F matrix (model determined by RAxML model picker) and a trimmed alignment containing the DBD and most of the LBD, beginning in helix 3. Support for particular nodes for maximum likelihood analyses was assessed with 1,000 bootstraps. For the Bayesian analysis, two independent analyses were performed with five chains run for five million generations and sampled every 500 generations. The first 1.25 million generations were discarded as burn-in. Log likelihood values were plotted and found to be asymptotic well before the burn-in fraction. Trees were visualized and illustrated with FigTree v1.1.2 (http://tree.bio.ed.ac.uk/software/figtree/).

## Results

### Nuclear receptors from the ctenophores Mnemiopsis leidyi and Pleurobrachia pileus

**Figure 1:**
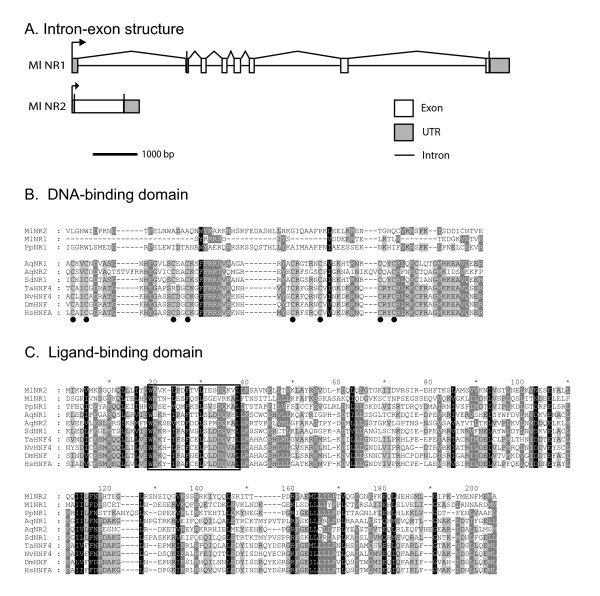
**Nuclear receptors from the ctenophores *Mnemiopsis leidyi *and *Pleurobrachia pileus***. **(A) **Intron-exon structure of the two nuclear receptors from *Mnemiopsis*. MlNR2 is a single exon gene. MlNR1 has a more complex intron-exon structure with eight exons, seven of which code for the inferred open-reading frame. **(B) **Alignment of the amino-terminal region of the ctenophore NRs with the DNA-binding domains of NRs from two sponges (*Amphimedon queenslandica *(Aq) and *Suberites domuncula *(Sd)), and *HNF4 *from *Trichoplax adhaerens *(Ta), *Nematostella vectensis *(Nv), *Drosophila melanogaster *(Dm), and *Homo sapiens *(Hs). The ctenophore proteins align poorly, including an absence of the conserved cysteines (indicated by black circles), and an optimized alignment contains insertions and deletions relative to the DBD of other animals. **(C) **Alignment of the ligand-binding domain from the same taxa as in (B). The ctenophore LBD is well-conserved, particularly the nuclear receptor signature motif spanning helix 3 and 4 (boxed).

BLAST queries of the *Mnemiopsis *genome and gene models resulted in two hits with significant similarity to the LBD of NRs from diverse metazoans. Assembled RACE products contained the complete open reading frame of each NR, as well as 5'- and 3'-UTR sequence (protein sequences are given in Additional file 3). *MlNR1 *is encoded by a transcript 1,408 bp long with an open reading frame of 268 amino acids. *MlNR1 *is composed of eight exons spanning almost 10 kb of genomic sequence, with the coding sequence on seven exons (Figure 1A). We identified a polyA tail 508 bp downstream from the stop codon and 156 bp of 5'-UTR. *MlNR2 *is encoded by a transcript 1,650 bp long with an open reading frame of 379 amino acids. *MlNR2 *is a single-exon gene with no intervening introns. The 3'-RACE sequence of *MlNR2 *contained a polyA tail 357 bp downstream of the stop codon. The 5'-RACE sequence contained 123 bp of UTR. By comparison, the assembled NR from *Pleurobrachia *ESTs had an open reading frame of 484 amino acids. We did not identify a polyA tail in the assembled transcript.

Neither of the two NRs from *Mnemiopsis *nor the single NR from *Pleurobrachia *contained a conserved zinc-finger DNA-binding domain (DBD) typical for NRs across the superfamily. The optimized alignment of the amino-terminal of the ctenophore NRs with the DBD from *HNF4 *from other animals suggests that this region in the ctenophore proteins contains very little conservation and is interrupted by insertions (Figure 1B). It was not possible to identify the conserved cysteines characteristic of zinc-finger motifs within the ctenophore sequences. The amino-terminal region of the ctenophore NRs failed to consistently match any protein or domain when BLASTed at NCBI. Despite the absence of a conserved DBD, each of the ctenophore NRs contains an LBD that aligned reasonably well with LBDs from other NRs (Figure 1C). Two of the ctenophore NRs (*MlNR2 *and *PpNR1*) have the NR signature motif (boxed region in Figure 1C), whereas *MlNR1 *has retained only a portion of this sequence. The relative conservation of the LBD in the ctenophore NRs was similar to that of NRs previously reported from two sponges [4,6,10] when compared with bilaterian *HNF4 *(approximately 30% identity, approximately 50% similarity).

### Developmental expression of Mnemiopsis nuclear receptors

**Figure 2:**
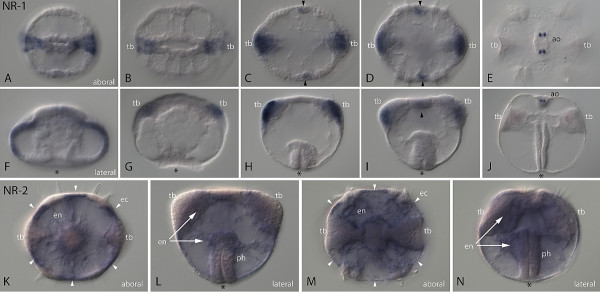
**Expression of *MlNR1 *(A-J) and *MlNR2 *(K-N) in embryos and juveniles of *Mnemiopsis***. **A-E **show aboral views of *MlNR1*, with F-J showing lateral views of the corresponding embryo. (A-B, **F-G**) *MlNR1 *expression in ectoderm of the future tentacle bulb (tb) in the gastrula and late gastrula stage. (C-D, **H-I**) Expression continues in ectoderm of future tentacle bulb as well as in additional domain along the sagittal plane (arrowhead) in the postgastrula. (E, **J**) *MlNR1 *expression is restricted to the apical organ (ao) in the cydippid stage. (**K-N**) *MlNR2 *is broadly detected in the endoderm (en) in developmental stages after gastrulation and in the cydippid. It is only in the most aboral part of the pharynx (ph). There are also additional domains in the ectoderm (white arrowheads).

Using *in situ *hybridization, we documented the developmental expression of *MlNR1 *and *MlNR2*. *MlNR1 *was first expressed during midgastrulation in the ectoderm in regions of future tentacle development (Figure 2A, B, F, G). Later in embryogenesis, expression continued in the same location, as well as in an additional adoral domain along the sagittal plane between the adpharyngeal comb rows (Figure 2C, D). In the cydippid stage, expression of *MlNR1 *was restricted to four discreet domains of the floor of the apical organ flanking the sagittal plane (Figure 2E, J). These were not associated with the balancing cilia that support the mineral containing lithocytes. *MlNR2 *was expressed broadly in the endoderm and parts of the ectoderm after gastrulation and in the cydippid (Figure 2K-N). Expression of *MlNR2 *was not observed earlier in gastrulation.

### Phylogenetic position of ctenophore NRs and distribution of NRs from early diverging metazoans

**Figure 3:**
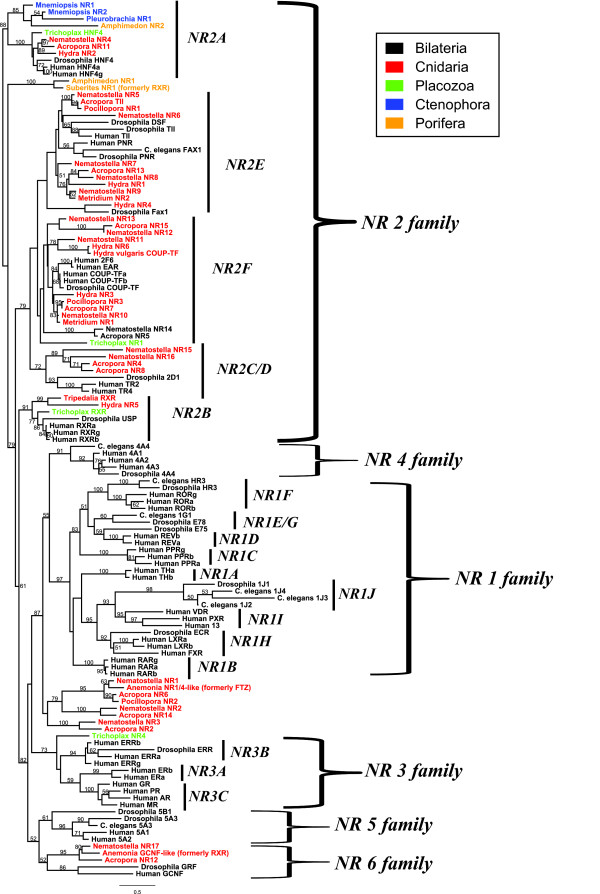
**Maximum likelihood tree of nuclear receptor superfamily**. Clades are annotated to family and subfamily based on current nomenclature for the NR superfamily [8]. Tree was rooted with the cluster containing the ctenophore sequences plus *HNF4 *from diverse animals. Values above nodes indicate percent of 1,000 bootstraps. Bootstrap values below 40 were removed.

Maximum likelihood analyses of the NRs from the selected taxa spanning the animal kingdom reproduced monophyletic relationships for all six recognized NR families (Figure 3). The bootstrap support for these families varied from 97% for NR family 1 to 52% for NR family 6. Previous studies have shown similarly low support for NR family 6 and for the node including this family and its sister family, NR family 5 [5,27,28]. Bayesian analyses resulted in a largely identical topology as the maximum likelihood analysis, with high posterior probabilities (0.93 to 1) for each NR family (Additional file 4).

The two NRs from *Mnemiopsis *and the one from *Pleurobrachia *cluster with the HNF4 subfamily (NR2A) and share greatest sequence similarity with *HNF4*s from other animals including *Trichoplax*, diverse cnidarians, and bilaterians. This cluster also contains a NR recently identified in the sponge *Amphimedon *[10]. The sponges *Amphimedon *and *Suberites *also contain a second NR-type identified by Larroux *et al. *[4] and Wiens *et al. *[6], respectively, that forms a sponge-specific NR cluster that groups between the HNF4 subfamily and all other nuclear receptors. These results suggest that ctenophores and sponges have representatives that most closely group with only one NR subfamily (NR2A) but that sponges contain an additional type of NR not present in any other species sampled to-date. Thus, ctenophores contain the most restricted NR complement of any species yet described.

For the placozoan *Trichoplax adhaerens*, we identified four NRs, consistent with results published earlier (Supplemental Figure S9.13 in [11]). *Trichoplax NR1 *grouped in NR family 2, with weak support in subfamily NR2F. Two additional NRs were strongly supported as orthologs to *HNF4 *(NR2A) and *RXR *(NR2B). *Trichoplax NR4 *was a strongly supported member of NR family 3. This result supports a previous report showing a NR 3 member from *Trichoplax *[12]. This earlier study concluded that this NR was likely an *ERR *ortholog. In our analysis, we found that this *Trichoplax *NR is a member of NR family 3, but with no particular relationship to any of the NR 3 subfamilies.

For the cnidarians, we analyzed NRs from five anthozoans, one cubozoan, and one hydrozoan. From the anthozoans, a previous study reported that the NR complement of *Nematostella vectensis *contained 17 NRs [3]. While most of the *Nematostella *NRs share well-supported orthologs with *Acropora*, orthologs of several of the *Nematostella *NRs have not yet been identified in *Acropora *or other corals. *Nematostella *sequences without clear *Acropora *counterparts are all sequences in subfamilies with coral orthologs, likely representing either paralogs potentially resulting from lineage-specific gene duplication or genes not yet sequenced from the coral. Three previously published NRs from the coral *Pocillopora damicornis *[29] grouped with *tailless *(NR2E), *COUP-TF *(NR2F), and the cnidarian specific NR1/4 clade. We identified two NRs from an EST collection for the anemone *Metridium*. These NRs grouped within family NR2E and 2F; one was orthologous to bilaterian *COUP-TF*s, and the other clustered with a cnidarian-specific NR radiation within family NR2E. We also included two previously published sequences from *Anemonia *[28] that grouped well with the anthozoan radiation of NR family 1/4 and a *GCNF*, despite their previous annotation as an *FTZ *and *RXR*, respectively. Together, the combined NR data show that anthozoans contain representatives of four of the five NR 2 subfamilies, with no ortholog of *RXR *(NR2B) in any species. Additionally, we identified three species (*Nematostella*, *Acropora*, and *Anemonia*) having an NR that groups most closely with *GCNF *(NR6), consistent with previous studies [5,10]. However, the support for these cnidarian genes with NR6 was modest and these genes may instead represent NR5/6 orthologs. Finally, in four anthozoan species, we observed at least one NR that groups in a cnidarian-specific cluster that was positioned as an outgroup to NR families 1 and 4, which only contain NRs from bilaterians. We originally identified three NRs from this group in *Nematostella*, leaving open the question as to whether these genes were unique to this anemone as a result of a species-specific duplication and divergence, or if these were more broadly represented in anthozoans. By including additional anthozoan genes, we identified well-supported coral orthologs to each *Nematostella *gene, bolstering the conclusion that this group is diverse within the class Anthozoa.

We identified six NRs from the recently completed genome of the hydrozoan *Hydra magnipapillata *[30]. One of these NRs (*HmNR6*) grouped strongly with one previously reported *COUP-TF *(NR2F) gene from the congener *H. vulgaris *[2]. These two NRs group in a separate cluster (with other cnidarian members of subfamily NR2F), but more distantly than with *COUP-TF*s from bilaterians and more closely related homologs from cnidarians, including an NR from *H. magnipapillata *(*HmNR3*). This suggests that these two NRs from two *Hydra *species are retained duplicates from an earlier cnidarian radiation in this family, a result consistent with that found by Escriva *et al. *[31]. The other NRs from *H. magnipapillata *were all supported as members of the NR2 family. *HmNR2 *was supported as an HNF4 family member. *HmNR1 *and *HmNR4 *group as members of the NR2E subfamily, although the position of *HmNR4 *varied within the NR2 family among analyses. The last NR from *H. magnipapillata *(*HmNR5*) grouped with strong support as an ortholog to *RXR*, grouping with *RXR*s from bilaterians and the placozoan *Trichoplax*. This NR is only the second *RXR *identified from the Cnidaria, with a previous report of a *RXR *from the cubozoan jellyfish *Tripedalia *[9]. Thus, *RXR *orthologs have only been reported from the medusozoan clade within the phylum Cnidaria. We did not identify an ortholog to NR family 1/4 or 6 in *H. magnipapillata*. Finally, we found no NR from *H. magnipapillata *or any anthozoan with support as a member of the NR family 3.

## Discussion

By characterizing the first NRs from the phylum Ctenophora and assembling NR sequences from diverse early diverging animals, we have gleaned new insights into the evolution of this superfamily of transcription factors. The ctenophore sequences in particular provoke new hypotheses about the origin of this gene superfamily and the evolution of the classical NR gene structure.

### Ctenophore NRs lacking a DBD

The zinc-finger DBD is typically the mostly conserved feature of NRs throughout the superfamily. The two NRs from *Mnemiopsis *and the one from *Pleurobrachia *have a conserved LBD, but all of these lacked a DBD typical of NRs. The two NRs from *Mnemiopsis *are supported as paralogs due to a duplication event somewhere in the ctenophore lineage. One of the *Mnemiopsis *NRs (*MlNR2*) is coded by a single exon; this is evidence of a potential retroposition event [32]. The ctenophore genes are the first NRs lacking a DBD from any non-bilaterian animal. We failed to identify any matches to a DBD elsewhere in the genome. The lack of a DBD and the potential position of ctenophores as the first branch from the animal tree suggest two competing hypotheses: the DBD was lost within the ctenophore lineage from an ancestral NR with both the DBD and LBD, or the ancestral NR contained only the LBD and the zinc-finger DBD was added later in animal evolution.

If the absence of the DBD is due to domain loss, it is likely to be broadly represented in ctenophores because *Mnemiopsis *and *Pleurobrachia *are members of separate orders - the Lobata and Cydippida, respectively [20]. The NRs reported from the three other early diverging phyla (Porifera, Placozoa, and Cnidaria) all contain both a DBD and an LBD. Within the bilaterians, NR domain structure has been modified in particular lineages, including duplication of the DBD in a novel protostome NR subfamily [33], loss of the DBD from vertebrate *DAX1 *(NR0B1) and *SHP *(NR0B2) [34,35], and loss of a conserved LBD in some NRs from insects (for example, *knirps *[36]) and nematodes (for example, *odr-7 *[37]). Thus, although loss of the DBD in ctenophores would be unique among early diverging animals, similar modifications of NR domains have occurred in other animals.

A more provocative but equally supported hypothesis for the absence of a canonical DBD in ctenophore NRs is that the ancestral NR contained only an LBD and the DBD was added later in animal evolution. This hypothesis is contingent on (1) the phylogenetic placement of ctenophores as the first branch of the animal tree, which has been shown in previous phylogenomic analyses [13,14] yet remains controversial [16,18], and (2) NRs from other ctenophore species lacking a zinc-finger DBD, like the distantly related species reported here. NRs are unique to animals, but several recent studies have shown "NR-like" genes in fungi [38]. These proteins (for example, *Oaf1 *and *Pip2 *from the yeast *Saccharomyces cerevisiae *[7]) have regions of structural similarity to animal NR LBDs but not zinc-finger DBDs, function as protein dimers, and bind fatty acids as ligands which regulate their function.

### Potential function of ctenophore nuclear receptors

Expression patterns of NRs from *Mnemiopsis *were consistent with NRs having a role in development. *MlNR1 *was expressed in spatially restricted portions of the ectoderm during gastrulation, corresponding to the position of the future tentacles. Subsequent expression in the cydippid stage was confined to the apical organ. However, *MlNR2 *was expressed broadly in the endoderm and portions of the ectoderm after gastrulation and in the cydippid stage. Previous studies with *HNF4 *in the sponge *Amphimedon *showed ubiquitous expression throughout development [10]. Spatial expression of *HNF4 *genes has not been reported from any cnidarian or the placozoan *Trichoplax*. However, *HNF4 *from the anemone *Nematostella *was expressed at high levels throughout developmental and adult stages, suggesting potential roles in both development and in normal cell physiology [5].

Nuclear receptors regulate the transcription of downstream genes, primarily through binding to specific DNA-responsive elements [39]. Due to the absence of a conserved DBD, the ctenophore NRs are unlikely to act as transcription factors by binding DNA-responsive elements typical of other NRs. The amino-terminal regions of these ctenophore NRs contain no conserved domains similar to other transcription factors; thus, we have no *a priori *expectation for what DNA motifs these proteins may bind (if any). Despite lacking a zinc-finger DBD, the mammalian NR *DAX-1 *binds DNA by recognizing hairpin structures [40] rather than through binding to specific sequence motifs. These ctenophore NRs may similarly regulate transcription of target genes in alternative ways by binding to non-canonical DNA sequences or secondary structures.

*DAX-1 *and *SHP *from vertebrates both function in molecular pathways as repressors. These NRs form dimers with other NRs, modulate the recruitment of cofactors, and repress transcriptional activity [41-43]. Whether the ctenophore NRs interact with other proteins or function as repressors requires additional research.

### Early divergence of the nuclear receptor superfamily

**Figure 4:**
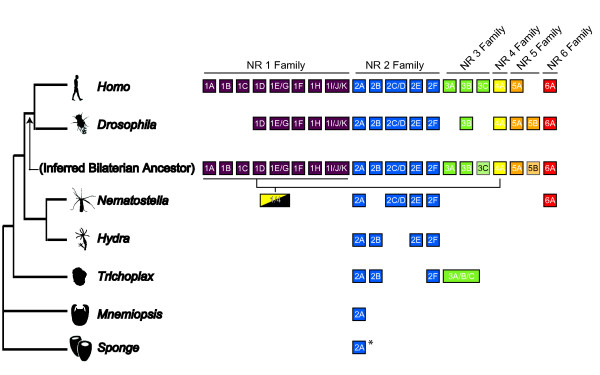
**Evolutionary diversification of the NR superfamily in animals**. At left is a cartoon of the metazoan tree showing the evolutionary relationships between ctenophores, sponges, placozoan, cnidarians, and bilaterians (that is, protostomes and deuterostomes). Due to current controversy about the branching order of the early diverging metazoans (see main text), the placement of lineages differs depending on particular analyses and thus an inference for the timing of origin and lineage-specific loss of particular NR families would vary. Colored boxes indicate a NR subfamily is represented by one or more genes for that species. The "inferred bilaterian ancestor" is based largely on a phylogenetic analysis conducted by Bertrand *et al. *[1]. However, two of these subfamilies are restricted to either protostomes (5B) or deuterostomes (3C) and we have shaded these and used black text to reflect a lack of conclusive support for the presence of these subfamilies in the bilaterian ancestor. Despite the absence of some genes in the *D. melanogaster *genome, studies of NRs from other protostomes (NR1A from *Schistosoma mansoni *[50] and NR3A from mollusks and annelids [51,52]) indicate that these subfamilies were present in the bilaterian ancestor and secondarily lost from *Drosophila*. Similarly, members of NR1B and 1C have been reported in mollusks and annelids [10], and thus are not restricted to deuterostomes. * The sponge *Amphimedon queenslandica *has two NRs, one that is supported as an ortholog to *HNF4 *(NR2A) and a second that groups between subfamily NR2A and the rest of the NR superfamily [see also [10]].

Previous reports on the evolution of the nuclear receptor superfamily have concluded that the diversification of NRs in bilaterians occurred through two major radiation events [1,44]. The first wave occurred prior to the bilaterian ancestor, due to the presence of all six NR families in both representative deuterostomes and protostomes ([1], Figure 4). A second stage of diversification occurred within the vertebrate lineage, with the expansion of particular subfamilies due to gene or genome duplication events (or example, the steroidogenic family NR3C). However, the early diversification of the NR superfamily prior to the bilaterian ancestor has not been clarified despite the critical importance of these events to the understanding of the emergence of the six NR families present at the divergence of protostomes and deuterostomes.

This new classification of nuclear receptors, which is based on the full complement of sequences from four early branching phyla as well as bilaterian sequences, allows us to begin reconstructing the evolutionary events that led to the diversification of this superfamily (Figure 4). The first two branches of the animal tree (sponge and ctenophore) both contain NRs that cluster with subfamily NR2A (*HNF4*) and are most similar to these sequences. Sponges also have a second NR positioned between *HNF4 *and the rest of the NR superfamily. Thus, these genes suggest that *HNF4 *represents our best expectation for which NR was present in the ancestral metazoan. Reciprocally, the limited diversity of NRs from sponges and ctenophores provides additional evidence that these two phyla represent the first two branches from the animal tree. The next branch in the metazoan tree is represented by the placozoan *Trichoplax *and suggests a partial radiation in NR family 2 with the addition of NR2B (*RXR*) and NR2F (*COUP-TF*) and the evolution of the first member of NR family 3 (as previously reported by [12]). The phylum Cnidaria is the closest sister group to the Bilateria from which NR sequences are currently available. From the two cnidarian species with complete genomes available (*Nematostella vectensis *and *Hydra magnipapillata*), we can infer that the cnidarian-bilaterian ancestor had a completely differentiated NR family 2, with NR2B (*RXR*) secondarily lost within the anthozoan lineage (see [5] for discussion). The class Anthozoa also has NRs that are best supported as NR family 6 (*GCNF*) and three class-specific paralogs that form an outgroup to NR families 1 and 4 in bilaterians. We hypothesize that the corresponding NRs were lost from *Hydra*, similar to a variety of other transcription factors (see [30]). Future characterization of NRs from other medusozoans, particularly members of the other two cnidarians classes Scyphozoa and Cubozoa, will allow additional testing of this hypothesis. Our data also support the hypothesis that the NR family 3 was lost early in the cnidarian lineage due to its presence in both *Trichoplax *and bilaterians. However, some phylogenetic analyses have suggested that Placozoa are the sister group to the Bilateria with the Cnidaria diverging from the stem earlier [for example, [18,45]]. If these are the correct phylogenetic relationships for these phyla, we would infer substantial NR loss from *Trichoplax*, including various members of NR family 2.

This represents the first study to classify NRs from each early animal lineage in a single analysis. From our analyses, a number of NR families and subfamilies differentiated in the time between the divergence of the cnidarian lineage and the origin of the bilaterians. Most striking is the diversification of the NR 1 family into numerous subfamilies, at least eight of which were present in the bilaterian ancestor. Gaining insight into the diversification that occurred between these ancestors will depend on characterizing the gene complement of species that diverged during this time period. The best current candidates are the acoelomorphs, which are supported as a closer sister taxa to the bilaterians than the cnidarians [13,46]. Indeed, studies of the Hox complement from these species have provided insightful intermediates for characterizing the emergence of the homeobox family of transcription factors [47,48].

By identifying NRs from these early branching metazoans with full genome sequences, we have additional power in characterizing the evolution of gene families, particularly since we are not inadvertently omitting genes missed in PCR-based surveys. For example, the original report of NRs from the coral *Acropora *identified ten NRs [3]. We queried newly published EST data from this species [23] and identified additional NRs that group in three families: the cnidarian-specific NR 1/4 family, NR2E, and NR2F. Because the *Acropora *sequences group with strong support with NRs from *Nematostella*, we expect that up to five more NRs are still not identified in this coral species.

With the additional cnidarian sequences and surveyed species, phylogenetic analyses suggest that NR evolution within the Cnidaria appears to have been a dynamic process with both gains (for example, duplication of *TR2/4, COUP-TF *anthozoans) and losses (NR family 6 and NR family 1/4 from either hydrozoans or ancestral lineage leading to the medusozoans, *RXR *from anthozoans). Two subfamilies in NR family 2 (NR2E and F) have independently radiated at some point in this phylum's history due to the presence of multiple cnidarian paralogs. *TR2/4 *(NR2C/D) most likely duplicated within the anthozoans due to the presence of two homologs in the anemone *Nematostella *and the coral *Acropora*. The most dramatic cnidarian-specific radiation is represented by the duplications of the group of NRs sister to NR families 1 and 4 (see above). Together, these data support a hypothesis that NRs have undergone a handful of independent radiations within the Cnidaria. For one class of cnidarians, the Cubozoa, there is only one published NR sequence [9] and for another class, the Scyphozoa, there are no published NRs. Identifying additional NRs in these two classes will provide much needed data for assessing when particular NRs were duplicated and lost within the cnidarians. Finally, we have no evidence that similar radiations have occurred in ctenophores, sponges, or placozoans. This supports the conclusion that the NR radiations in the Cnidaria are unique among the early diverging animal lineages.

## Conclusion

In this study, we have identified two NRs from the *Mnemiopsis leidyi *genome and one NR from *Pleurobrachia pileus*. All three ctenophore NRs contain the conserved LBD but lack a conserved zinc-finger DBD, a domain that is conserved across all reported NRs in animals (with the exception of two genes unique to vertebrates). By applying a phylogenomic approach using NR sequences from organisms throughout the animal kingdom, we showed that ctenophores and sponges contain representatives of the same subfamily (NR2A), suggesting that the original NR was most similar to *HNF4*. The absence of the DBD from ctenophores may reflect an ancestral NR domain structure or a lineage-specific loss of this domain from an ancestral NR that contained both the DBD and LBD. Through analysis of NR family and subfamily representation in representative taxa, we conclude that the rate of diversification for the NR superfamily was fairly modest in the early diverging animals, similar to other gene families [49]. Additionally, several subfamilies underwent separate radiations in the phylum Cnidaria. Future work aimed at characterizing the function of the NRs from these early diverging phyla will enable tests of hypotheses regarding conserved and novel functions of members of this critical superfamily of transcription factors.

## Abbreviations

BLAST: basic local alignment search tool; cDNA: complementary DNA; COUP-TF: chicken ovalbumin upstream promoter transcription factor; DAX-1: dosage-sensitive sex reversal-adrenal hypoplasia congenital critical region on the X chromosome; DBD: DNA-binding domain; DNA: deoxyribonucleic acid; ERR: estrogen related receptor; EST: expressed sequence tag; FTZ: fushi tarazu; GCNF: germ cell nuclear factor; HMM: hidden Markov model; HNF4: hepatocyte nuclear factor 4; LBD: ligand-binding domain; NR: nuclear receptor; PCR: polymerase chain reaction; RACE: rapid amplification of cDNA ends; RNA: ribonucleic acid; RXR: retinoid "X" receptor; SHP: small heterodimerization partner; UTR: untranslated region.

## Competing interests

The authors declare that they have no competing interests.

## Authors' contributions

AMR and AMT designed and conceived the study and drafted the manuscript. AMR also isolated *Mnemiopsis *nuclear receptors, performed gene annotation, and performed phylogenetic analyses. JFR identified *Mnemiopsis *nuclear receptors from the genomic assembly and contributed to phylogenetic analyses. KP isolated *Mnemiopsis *DNA and RNA and performed *in situ *hybridizations. JCM assembled the *Mnemiopsis *genome. ADB and MQM participated in the design of the study. All authors read and approved the final manuscript.

## Supplementary Material

Additional file 1 **Primers used for 5- and 3-prime RACE of *Mnemiopsis leidyi *NRs**. Click here for file

Additional file 2 **Nuclear receptors from early diverging taxa used for phylogenetic study of nuclear receptor superfamily**. Table showing gene names and accession numbers Click here for file

Additional file 3 **Protein sequences for NRs from *Mnemiopsis leidyi *and *Pleurobrachia pileus***. Click here for file

Additional file 4 **Bayesian analysis of NR superfamily**. Tree was constructed using the identical alignment used for the maximum likelihood analysis presented in Figure 3. Clades are annotated to family and subfamily based on current nomenclature for the NR superfamily [8]. This tree is the consensus of four independent runs and was rooted with the cluster containing the ctenophore sequences plus *HNF4 *from diverse animals. Values above nodes indicate Bayesian support values. Posterior probabilities below 0.7 were removed. Click here for file
