# Supplementary material for: Nuclear receptors from the ctenophore Mnemiopsis leidyi lack a zinc-finger DNA-binding domain: lineage-specific loss or ancestral condition in the emergence of the nuclear receptor superfamily?
Source: EvoDevo. 2011 Feb 3;2:3. doi: 10.1186/2041-9139-2-3 (PMC3038971; doi:10.1186/2041-9139-2-3)
Supplement: Additional file 1 — Primers used for 5- and 3-prime RACE of Mnemiopsis leidyi NRs. [file 2041-9139-2-3-S1.PDF]

Additional File 1. Primers used for 5- and 3-prime RACE of *Mnemiopsis leidyi* NRs.

MlNR1

Primer set 1:
LEFT PRIMER TTACAATCCCAGCGAAGGAT
RIGHT PRIMER AAGGGGAGGGTAGAGAAGCA

Primer set 2:
LEFT PRIMER TACCAACGACGAGGAACTTG
RIGHT PRIMER GCTTAACCTCCAGCTTGTCG

MlNR2

Primer set 1:
LEFT PRIMER TGGAGGAGCTAAAGCGAGAG
RIGHT PRIMER GGAAGCTTAACTGCCCACTG

Primer set 2:
LEFT PRIMER TGGTCAAAACCAGCTTCTCC
RIGHT PRIMER CCCTCTGTATGTGGGTTGAAG
