# Supplementary material for: Nuclear receptors from the ctenophore Mnemiopsis leidyi lack a zinc-finger DNA-binding domain: lineage-specific loss or ancestral condition in the emergence of the nuclear receptor superfamily?
Source: EvoDevo. 2011 Feb 3;2:3. doi: 10.1186/2041-9139-2-3 (PMC3038971; doi:10.1186/2041-9139-2-3)
Supplement: Additional file 2 — Nuclear receptors from early diverging taxa used for phylogenetic study of nuclear receptor superfamily. Table showing gene names and accession numbers [file 2041-9139-2-3-S2.PDF]

Additional File 2. Nuclear receptors from early diverging taxa used for phylogenetic study of nuclear receptor superfamily. Joint Genome Institute (JGI) numbers refer to most-complete gene models available at time of this study.

Species	NR Name	Accession/ID	Source
Mnemiopsis leidyi	MlNR1	Table S3	This study
	MlNR2	Table S3	This study
Pleurobrachia pileus	PpNR1	Table S3	This study
Amphimedon queenslandica	AqNR1	ACA04755	[4]
	AqNR2	ADK78987	[10]
Suberites domuncula	SdRXR*	CAD57002	[6]
Trichoplax adhaerens	TaNR1	XP_002109806	[11]
	TaNR2	XP_002109459	"
	TaNR3	XP_002115810	"
	TaNR4	XP_002117374	"
Hydra vulgaris	HvCOUP	AAU11312	[2]
Hydra magnipapillata	HmNR1	XP_002154441	[30]
	HmNR2	XP_002159483	"
	HmNR3	XP_002159396	"
	HmNR4	XP_002156561	"
	HmNR5	XP_002165675	"
	HmNR6	XP_002167930	"
Tripedalia cystophora	TcRXR	AAC80008	[9]
Metridium senile	MsNR1	FC846837	NCBI
	MsNR2	FC847366	NCBI
Anemonia sulcata	AsFTZ	AAB68693	[31]
	AsRXR	AAB68695	"
Acropora millepora	AmTLL	AAL29193	[3]
	AmNR2	AAL29194	"
	AmNR4	AAL29196	"
	AmNR5	AAL29198	"
	AmNR6	AAL29199	"
	AmNR7	AAL29200	"
	AmNR8	AAL29201	"
	AmNR9 (11)**	AAL29202	"
	AmNR10 (12)**	AAL29203	"
	AmNR13	EZ031572	This study
	AmNR14	EZ029516	This study
	AmNR15	EZ015636	This study
Pocillopora damicornis	PdNR1		[29]
	PdNR2		"
	PdNR3		"
Nematostella vectensis	NvNR1	JGI: 101676	[5]
	NvNR2	JGI: 99425	"

	NvNR3	JGI: 108851	“
	NvNR4	JGI: 89471	“
	NvNR5	JGI: 114090	“
	NvNR6	JGI: 183874	“
	NvNR7	JGI: 169225	“
	NvNR8	JGI: 99425	“
	NvNR9	JGI: 247458	“
	NvNR10	JGI: 189134	“
	NvNR11	JGI: 242271	“
	NvNR12	JGI: 165424	“
	NvNR13	JGI: 203423	“
	NvNR14	JGI: 202735	“
	NvNR15	JGI: 167880	“
	NvNR16	JGI: 244121	“
	NvNR17	JGI: 218255	“

* Phylogenetic analyses support this gene as a sponge-specific NR, not a RXR ortholog as originally described in Wiens et al. [6].

** Number in parentheses indicates new number assigned based on extended sequence matching a previously reported NR [3].
