# Supplementary material for: Nuclear receptors from the ctenophore Mnemiopsis leidyi lack a zinc-finger DNA-binding domain: lineage-specific loss or ancestral condition in the emergence of the nuclear receptor superfamily?
Source: EvoDevo. 2011 Feb 3;2:3. doi: 10.1186/2041-9139-2-3 (PMC3038971; doi:10.1186/2041-9139-2-3)
Supplement: Additional file 3 — Protein sequences for NRs from Mnemiopsis leidyi and Pleurobrachia pileus. [file 2041-9139-2-3-S3.PDF]

Additional File 3. Protein sequences for NRs from *Mnemiopsis leidyi* and *Pleurobrachia pileus*.

>MlNR1
MVSYRRRSDIYSVDDEKRTELRTLRTEDGKVETVRVHPLVPRWPEEYVDGQYSV
SNEFMSPQQFPPTITHIDSGKIVNSGKKQLTELHQWATNLESFQSLSGEVRKALFT
NSITLLLLLLKFAFKSSKASAKQIQDGVKSCYNPSEGSSEQVVQKVGKLILDKLVSP
LQSLITNDEELELFMAIILFDPSCETLNDESERVKQFRQETCDKLEVKLNDKGEKL
SDLLLLYPPLCTVRSAIIEHLSLVELIGEASDLRNNARDMMTNLLG

>MlNR2
MYPRPFDDDEEAWREAWTCKFSSKSHTLASWLVQILNKGVLGHWIDPKNGTFE
LNWADAAQNWYKAKKRHSRFEDASHLLHKGIQAAFPKLEELKRENTGHQCVR
RSFKISDDICNTVEAIVVGTSNKERKRCKCNNSQYLQFQTMWPLSEHLLGFDTPI
KVTPIRDNAYQHIALVPSDSPTNEEMIKWVMKSGQNQLLLIYQWAVKLPDFTVLI
ESDQKVLLKSAVNELLTVKLAYRSVDLKDQLQLGTGKIIDVRSIREHFTKSLAMS
VVKNIVSTLNELKVNESEFALLQQIILFNPHTEGLRSNSIQGVKSSRRKIYQQLSRI
TTPDRFAEMLLILPTVQGIGNEFKEQLNEHSMLGIPKYMENFMEMANFKQKL

>PpNR1
MECGGLPFITRPASLKREHPPSSPGSSESYFDEDGWRKAWQVKFKYGKSSNLSV
WLIDRLTRSIGGRWLSMEDGRFELEWIDTANAWYAERQRRSKSSQSTHLLYKAI
MAAFPNIAEESSEKNKHIFVKRSFKLPNELCEEVREIVIGTGTKRAALHKTIATIAG
PASLAEITGPRYLVDNMGVVLPKAQYKVPDHLMKDFMSAVSSTSQAITEENSPA
PVTKLVSAWNLDLPTENSGVVNTSRCTTKTIPPNEVGSPPISPNFLLDGPSPTRYTS
SQEATFEQICEYAKQQLEIIHKWASEMQMFKSLSVSDQKALLTSTAPEILVFSICC
FSVQNTCGLRLDNLILDKDLVISRTDRQYDMAIRNIVSFIMDTIVIPLKDLGTDRFE
LSRLRRIILFNSTTKANYQSLKDPEEVRLIRKTEHTLLQKYNEGKPGRIGELLLLLP
PLTTAGNLFIEHLHLEKLVGDTFENLVAAELLVAMGSG
