# Supplementary figures and images for: Nuclear receptors from the ctenophore Mnemiopsis leidyi lack a zinc-finger DNA-binding domain: lineage-specific loss or ancestral condition in the emergence of the nuclear receptor superfamily?
Source: EvoDevo. 2011 Feb 3;2:3. doi: 10.1186/2041-9139-2-3 (PMC3038971; doi:10.1186/2041-9139-2-3)

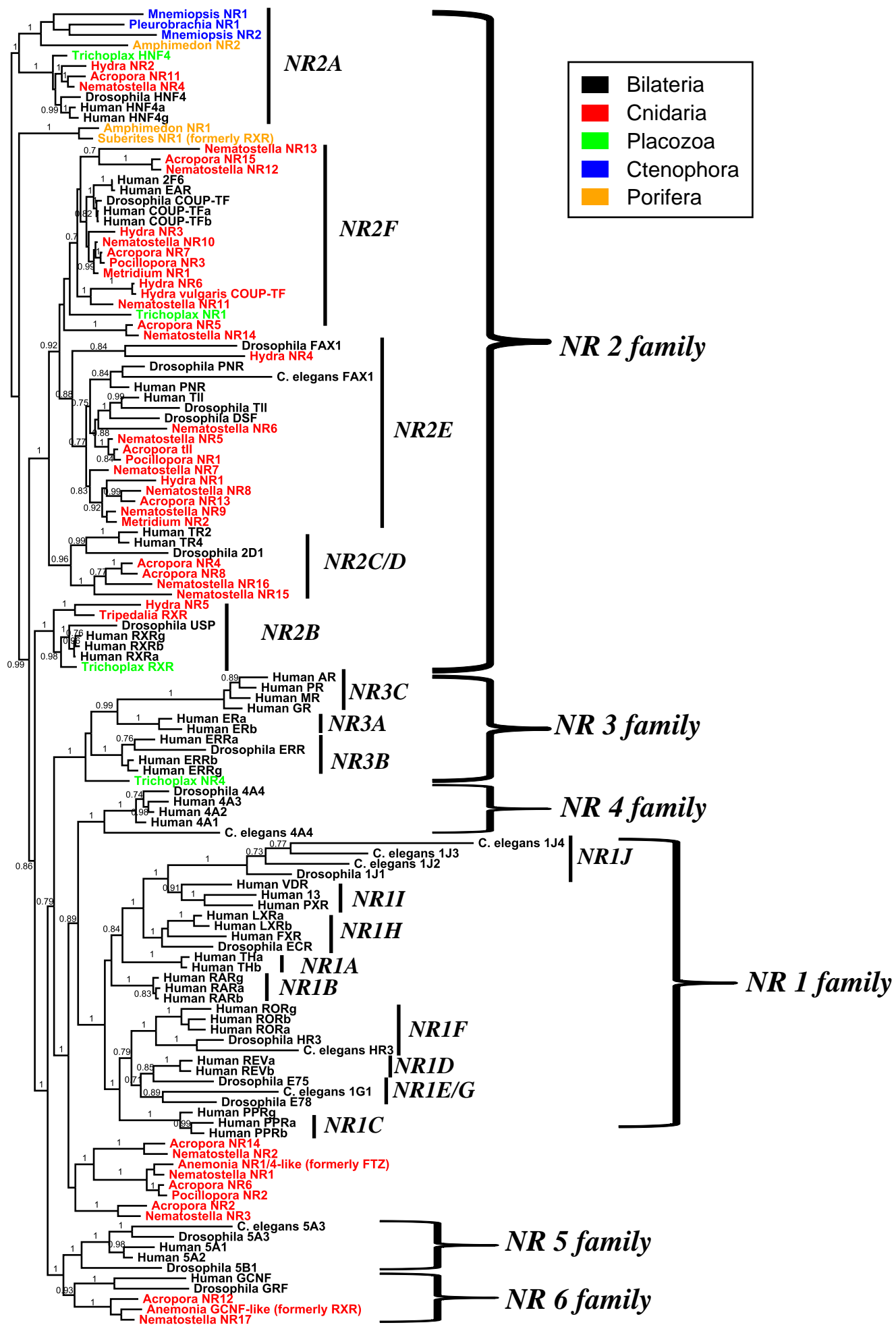

Supplement: Additional file 4 — Bayesian analysis of NR superfamily. Tree was constructed using the identical alignment used for the maximum likelihood analysis presented in Figure 3. Clades are annotated to family and subfamily based on current nomenclature for the NR superfamily [8]. This tree is the consensus of four independent runs and was rooted with the cluster containing the ctenophore sequences plus HNF4 from diverse animals. Values above nodes indicate Bayesian support values. Posterior probabilities below 0.7 were removed. [file 2041-9139-2-3-S4.PDF]
